# Screening Plasma Exosomal RNAs as Diagnostic Markers for Cervical Cancer: An Analysis of Patients Who Underwent Primary Chemoradiotherapy

**DOI:** 10.3390/biom11111691

**Published:** 2021-11-14

**Authors:** Oyeon Cho, Do-Wan Kim, Jae-Youn Cheong

**Affiliations:** 1 Department of Radiation Oncology, Ajou University School of Medicine, Suwon 16499, Korea; 2 Ajou Translational Omics Center, Ajou University School of Medicine, Suwon 16499, Korea; kdowan@ajou.ac.kr (D.-W.K.); jaeyoun620@gmail.com (J.-Y.C.); 3 Department of Gastroenterology, Ajou University School of Medicine, Suwon 16499, Korea; 4 Human Genome Research and Bio-Resource Center, Ajou University Medical Center, Suwon 16499, Korea

**Keywords:** cervical cancer, plasma exosomes, non-coding RNA, mRNA, cancer screen, radiation therapy

## Abstract

This preliminary study aimed to screen non-coding RNAs (ncRNAs) from plasma exosomes as a new method for cervical cancer diagnosis. Differentially expressed RNAs were initially selected from among a group of 12 healthy individuals (normal group) and a pretreatment group of 30 patients with cervical cancer (cancer group). Then, we analyzed the association between an ncRNA-mRNA network and cancer using ingenuity pathway analysis after secondary selection according to the number and correlation of mRNAs (or ncRNAs) relative to changes in the expression of primarily selected ncRNAs (or mRNAs) before and after chemoradiotherapy. The number of RNAs selected from the initial RNAs was one from 13 miRNAs, four from 42 piRNAs, four from 28 lncRNAs, nine from 18 snoRNAs, 10 from 76 snRNAs, nine from 474 tRNAs, nine from 64 yRNAs, and five from 67 mRNAs. The combination of miRNA (miR-142-3p), mRNAs (*CXCL5, KIF2A, RGS18, APL6IP5*, and *DAPP1*), and snoRNAs (SNORD17, SCARNA12, SNORA6, SNORA12, SCRNA1, SNORD97, SNORD62, and SNORD38A) clearly distinguished the normal samples from the cancer group samples. We present a method for efficiently screening eight classes of RNAs isolated from exosomes for cervical cancer diagnosis using mRNAs (or ncRNAs) altered by chemoradiotherapy.

## 1. Introduction

The age-standardized incidence rate (ASR) in 100,000 for cervical cancer in South Korea was 8.7% in 2017, which was a significant improvement over the rate of 18.6% in 1999 [1,2]. This decrease in ASR is thought to be related to the establishment of regular health check-ups and dissemination of human papillary virus vaccines. However, unlike the overall ASR improvement, the ASR of 25–29-year-olds gradually increased from 3.6% in 2000 to 6.5% in 2011 [2]. The incidence of cervical cancer has decreased in 60–70-year-old individuals but increased in those aged between 20–30 years, and this age-dependent incidence has become clearer as the level of national development increases [3]. This pattern may be attributed to the active participation of young women in the national health check-up program, the high intake of junk food and smoking habits in young women, and sexual intercourse at younger ages [4,5]. In Korea, a cervical cytology test is currently performed every three years for women over the age of 20 years [6]. However, not only is the pap smear as a screening test a potential source of fear and shame, its sensitivity can also be as low as 50% [7]. Although this limitation can be overcome by the high sensitivity of an HPV test, 12.7% of squamous cell carcinoma (SCC) and 15–38% of adenocarcinoma (AC) globally are not related to HPV [8]. In addition, the sensitivity of the pap smear test can further decrease depending on the skill level of the physician and the examination environment. Therefore, there is an unmet need for cervical cancer screening methods that use blood or body fluids.

Exosomes are extracellular vesicles of endosomal origin, with diameters of 30–100 nm that contain various biomolecules such as nucleic acids, lipids, and proteins [9]. All types of cells perform intercellular communication through exosomes to maintain cellular homeostasis via an inflammatory response [10]. In addition, cancer-associated exosomes are thought to play an important role in tumor promotion by inducing angiogenesis, remodeling the extracellular matrix, and impairing immune cell function [11]. In particular, there are various studies on the association among exosomal non-coding RNA (ncRNA), messenger RNA (mRNA), and various cancers such as cervical, pancreatic, prostate, and lung cancers [12,13,14,15,16]. The screening methods used in these studies depended on differentially expressed gene (DEG) analysis and preclinical studies using cancer cells, with the screening range confined to a few specific RNA classes such as microRNA (miRNA), small nucleolar RNA (snoRNA), and messenger RNA (mRNA). A previous study analyzed a network of mRNAs relative to a log_2_-fold change (log_2_FC) in the expression of candidate miRNAs before and two weeks after cisplatin-based concurrent chemoradiotherapy (CCRT) to predict clinical results in patients with cervical cancer, and suggested their potential biological functions using ingenuity pathway analysis (IPA) [17]. This study suggested that biological functions or diseases can be detected from the association between ncRNAs and mRNAs that are altered by clinical treatments if the exosome sequencing data are paired with the specific treatment.

The current pilot study aimed to screen plasma exosomal RNAs for cervical cancer diagnosis from seven classes of ncRNAs (miRNA, piwi-interacting RNA (piRNA), long non-coding RNA (lncRNA), snoRNA, small nuclear RNA (snRNA), transfer RNA (tRNA), yRNA) and mRNA. The ncRNAs or mRNAs were initially selected through the DEG analysis of healthy individuals and patients with cervical cancer and then further tested by constructing a network that consisted of mRNAs or ncRNAs that were associated with changes in the expression of the initially selected ncRNAs (or mRNAs) in individual pairwise comparisons of plasma exosomal RNAs before and after CCRT.

## 2. Materials and Methods

### 2.1. Blood Samples and Clinical Data

Two sets of 5–10 mL blood samples were collected from 30 patients diagnosed with stage IB-IVB cervical cancer and treated with CCRT at the Department of Radiation Oncology at Ajou University from June 2018 to March 2020. These samples were stored at the Biobank at Ajou University Hospital, which is a member of the Korea Biobank Network. Sixty samples in total were acquired before treatment and after the second week of CCRT. Blood plasma samples (3 mL) from 12 healthy individuals matched for sex and age were obtained from the Biobank at Ajou University Hospital (institutional review board approval number: BMR-EXP-20-428). Plasma exosomal RNA sequencing was conducted by Macrogen (www.macrogen.com, Supplementary Methods). The diagnosis of all patients was histologically confirmed via biopsy. Regional lymph node metastasis and distant metastasis were evaluated using magnetic resonance imaging and positron emission tomography-computed tomography. Detailed treatment and follow-up procedures were previously reported for most of the current patients [17]. Clinical data, such as information regarding age, 2018 International Federation of Gynecology and Obstetrics stage, pathology, radiation therapy (RT) field, pretreatment hemoglobin levels, pretreatment absolute lymphocyte count (ALC), ALC after two weeks of CCRT (ALC2), and the levels of pretreatment tumor markers SCC antigen and cytokeratin fragment 21-1 were collected retrospectively from electronic medical records.

### 2.2. Screening Process

**Figure 1:**
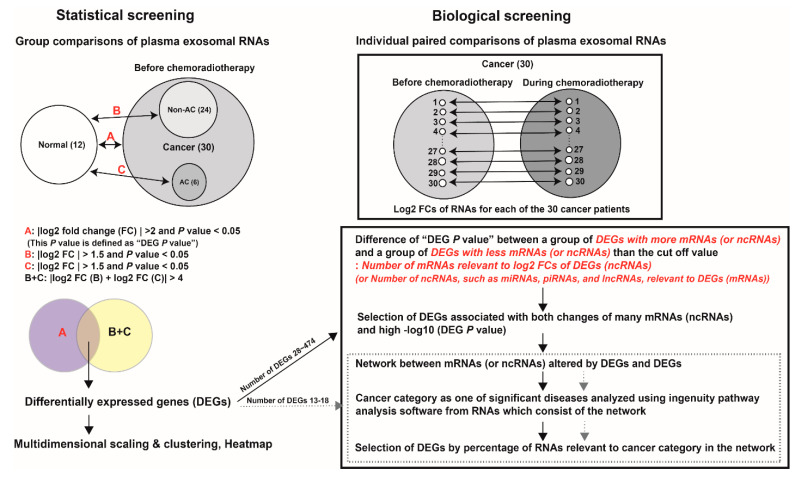
A plasma exosomal RNA screening method for cancer diagnosis that consists of a statistical screening phase followed by a biological screening phase.

The screening process of seven classes of ncRNAs and mRNAs from plasma exosomes for cervical cancer diagnosis is presented in Figure 1. The process was divided into statistical and biological screening phases.

#### 2.2.1. Statistical Screening

The expression of plasma exosomal ncRNAs and mRNA was compared between a group of 12 samples from healthy individuals (normal group) and a group of 30 samples from patients with cervical cancer before CCRT (cancer group). RNAs with both |log_2_FC| > 2 and *p*-values < 0.05 were selected from DEG analysis (A). The *p*-value of (A) was defined as the “DEG *p*-value”. The expression of plasma exosomal ncRNAs and mRNA was compared between the normal group and a group of 24 samples from patients with cervical SCC or unclassified carcinoma before treatment (non-AC group). RNAs with |log_2_FC| >1.5 and *p*-values < 0.05 were selected from DEG analysis (B).

The expression of plasma exosomal ncRNAs and mRNA was compared between the normal group and a group of six samples from patients with cervical adenocarcinoma or adeno-squamous cell carcinoma before treatment (AC group). RNAs with |log_2_FC| >1.5 and *p*-values < 0.05 were selected from DEG analysis (C). The significant DEGs were primarily selected when the RNAs of (A) were simultaneously included in those with |log_2_FC (B) + log_2_FC (C)| > 4. These results were visualized using volcano plots.

#### 2.2.2. Biological Screening

We calculated the log_2_FC values of plasma exosomal RNAs from 30 patient samples taken two weeks after CCRT, which were compared with those collected before CCRT to screen for secondary biological functions of the initially selected DEGs. If the number of DEGs was less than 20 for miRNA and snoRNA, we analyzed the extent to which the network of mRNAs relative to the log_2_FC DEG(s) was associated with cancer category as described by IPA, and calculated the percentage of RNAs relative to the cancer category in the network. If the number of DEGs was more than 20, the DEGs were divided into two groups to maximize the difference in “DEG *p*-values” according to number of mRNAs associated with the log_2_FCs in the selected ncRNAs (e.g., lncRNA, piRNA, snRNA, tRNA, and yRNA). When a class of selected RNAs was mRNA, we used the number of miRNAs, piRNAs, and lncRNAs relative to the identified DEGs. We performed a network analysis and IPA after screening the DEGs satisfied with many mRNAs or ncRNAs, and high −log_10_(DEG *p*-value) based on this grouping.

### 2.3. Statistical Analysis of Differential RNA Expression

Raw data (i.e., the reads for each RNA) were normalized by TMM (trimmed mean of M value) using edgeR. For pre-processing, the RNAs undetected in over 12 of 42 samples and those undetected in over 30 of 60 samples were filtered during DEG analysis for statistical and biological screening, respectively.

### 2.4. Multidimensional Scaling and Heatmap Construction

Classical multidimensional scaling (MDS) of all 42 samples calculated by the cmdscale function was visualized by scattered data partitioned into two or three groups using k-means clustering. A hierarchical clustering heatmap was plotted using the pheatmap function. In MDS and heatmap analysis, log_2_ count per million (CPM) values from normalized read counts were used for each RNA class from the statistical screening, while those from raw read counts were applied to optimally integrate the different classes of RNAs that resulted from the screening process.

### 2.5. Network Analysis

We formed a network using Prim’s algorithm for the minimum spanning tree in the igraph package for R. Edges are presented in red and blue, which indicate positive and negative correlations, respectively.

### 2.6. Ingenuity Pathway Analysis

The most significant diseases and bio-functions were analyzed using the IPA software (Qiagen, https://www.qiagenbioinformatics.com/products/ingenuity-pathway-analysis) and selected based on the activated Z-scores of the downstream effects of the analysis [18]. Positive and negative Z-scores indicated the induced and inhibited functional activities, respectively.

### 2.7. Receiver Operative Characteristic Analysis

We visualized receiver operative characteristic (ROC) curves and calculated the area under the curves, sensitivities, and specificities using the Epi and pROC packages.

### 2.8. Table and Boxplots

Continuous variables between groups were compared using the *t*-test or Wilcoxon rank-sum test according to a Shapiro–Wilk test, while categorical variables were compared using Fisher’s exact test or the chi-square test (Table 1). Statistical analysis for logarithmic values of reads per million mapped reads (RPM) or reads per kilobase per million mapped reads (RPKM) between groups was performed using the Wilcoxon rank-sum test or Kruskal-Wallis test in all boxplots. Data analysis and visualization were performed using R version 4.1 (https://www.r-project.org).

## 3. Results

### 3.1. Clinical Characteristics

Table 1 shows the clinical characteristics of the cancer group (*n* = 30). Patients with cervical cancer confined within the pelvis (stage IB–IIIC1) made up 66.7% of the cohort, while those that had extra-pelvic metastases made up 33.3% of the group. Of the 24 non-AC patients, 23 (95.8%) had SCC pathology.

### 3.2. Statistical Screening

**Figure 2:**
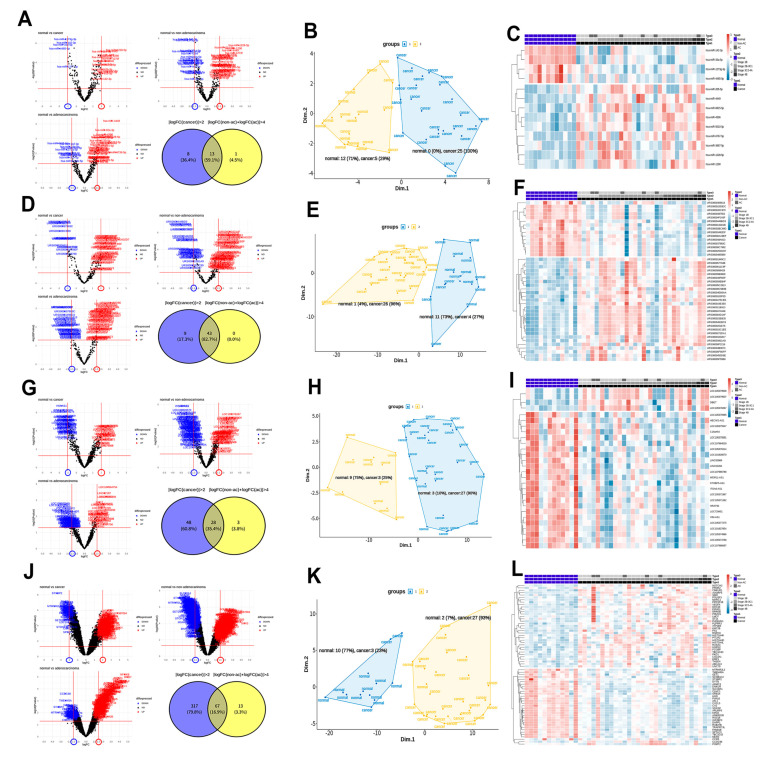
Statistical screening of miRNAs, piRNAs, lncRNAs, and mRNAs as markers for cervical cancer diagnosis. (**A**) Volcano plots and a Venn diagram of the selected 13 miRNAs. (**B**) A clustered multidimensional scaling (MDS) scatter plot for 42 samples using the 13 selected miRNAs. (**C**) A heatmap of the 13 miRNAs for the normal and cancer groups. (**D**) Volcano plots and a Venn diagram of the selected 42 piRNAs. (**E**) A clustered MDS scatter plot for the 42 samples using the 42 piRNAs. (**F**) A heatmap of the 42 piRNAs for the normal and cancer groups. (**G**) Volcano plots and a Venn diagram of the selected 28 lncRNAs. (**H**) A clustered MDS scatter plot for 42 samples using the 28 lncRNAs. (**I**) A heatmap of 28 lncRNAs for the normal and cancer groups. (**J**) Volcano plots and a Venn diagram of the selected 67 mRNAs. (**K**) A clustered MDS scatter plot for the 42 samples using 67 mRNAs. (**L**) A heatmap of the 67 mRNAs for the normal and cancer groups.

We selected 13 miRNAs, 43 piRNAs, 28 lncRNAs, and 67 mRNAs from three DEG analyses, as shown in Figure 2A,D,G,J (normal vs. cancer group, normal vs. non-AC group, and normal vs. AC group). The MDS scatter plots and k-means clustering of the selected RNAs divided 42 samples into two groups that had both sensitivity and specificity for cancer diagnosis in each of the four RNA classes (Figure 2B,E,H,K). The sensitivity and specificity of miRNAs were 83% (predicted 25 of 30 patients) and 100%, piRNAs were 87% (predicted 26 of 30 patients) and 92% (predicted 11 of 12 healthy individuals), lncRNAs were 90% (predicted 27 of 30 patients) and 75% (predicted 9 of 12 healthy individuals), and mRNAs were 90% (predicted 27 of 30 patients) and 83% (predicted 10 of 12 healthy individuals), respectively. Heatmaps of DEG expression levels for the four groups showed differences between the normal and cancer groups, but did not display differences in disease stage or pathology (Figure 2C,F,I,L).

**Figure 3:**
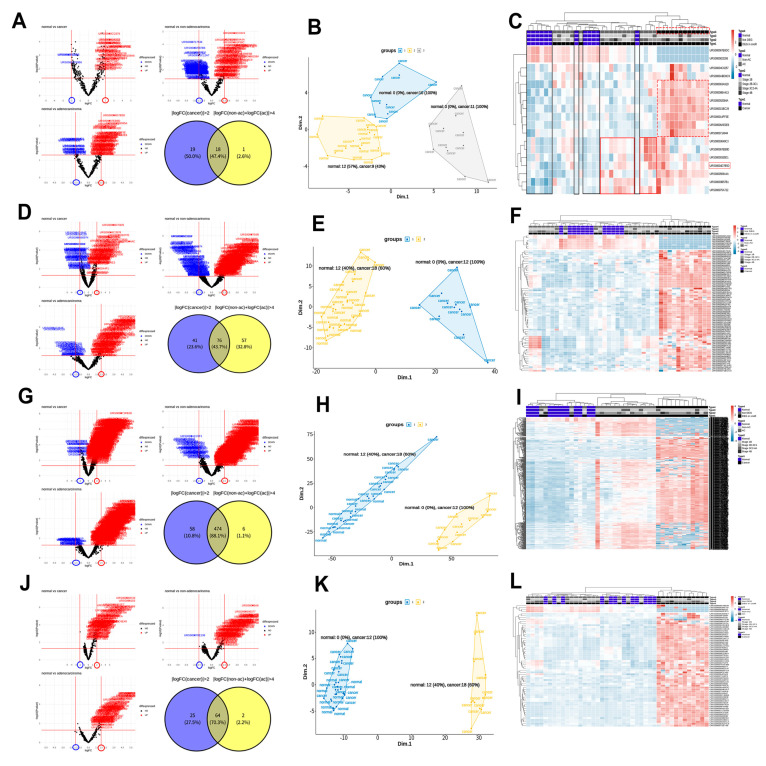
Statistical screening of exosome snoRNAs, snRNAs, tRNAs, and yRNAs for the diagnosis of cervical cancer. (**A**) Volcano plots and a Venn diagram of 18 selected snoRNAs. (**B**) A clustered multidimensional scaling (MDS) scatter plot for 42 samples using the 18 snoRNAs. (**C**) A heatmap of the 18 snoRNAs in the normal and cancer groups. (**D**) Volcano plots and a Venn diagram of the selected 76 snRNAs. (**E**) A clustered MDS scatter plot of the 42 samples using 76 snRNAs. (**F**) A heatmap of the 76 snRNAs measured in the normal and cancer groups. (**G**) Volcano plots and a Venn diagram of the selected 474 tRNAs. (**H**) A clustered MDS scatter plot of the 42 samples using 474 tRNAs. (**I**) A heatmap of the 474 tRNAs in the normal and cancer groups. (**J**) Volcano plots and a Venn diagram of the selected 64 yRNAs. (**K**) A clustered MDS scatter plot of the 42 samples using the 64 yRNAs identified. (**L**) A heatmap of the 64 yRNAs in the normal and cancer groups.

A total of 18 snoRNAs, 76 snRNAs, 474 tRNAs, and 64 yRNAs were selected from three DEG analyses, as presented in Figure 3A,D,G,J (normal vs. cancer group, normal vs. non-AC group, and normal vs. AC group). The MDS scatter plot and k-means clustering of selected snoRNAs identified three groups with a sensitivity of 70% (predicted 21 of 30 patients) and specificity of 100% for cancer diagnosis (Figure 3B). In Figure 3C, the heatmap of snoRNA expression in 42 samples shows two groups of snoRNAs that could distinguish between the normal and cancer groups. A group of snoRNAs (Figure 3C; red dotted line) was homogeneously overexpressed in 12 patients within the cancer group, while a group of snoRNAs (Figure 3C; red solid line) was nonhomogeneously overexpressed in the other 12 patients within the cancer group. We defined the former group as the ***DEG in snoRNA***. MDS scatter plots and k-means clustering using selected snRNAs, tRNAs, and yRNAs divided 42 samples into two groups with a sensitivity of 40% (predicted 12 of 30 patients) and specificity of 100% for cancer diagnosis for all three types of RNAs (Figure 3E,H,K). The heatmaps for these three groups showed that the 12 patients with ***DEG in snoRNA*** were classified into the same group in a column hierarchical clustering through the expression of selected RNAs (Figure 3F,I,L).

### 3.3. Biological Screening

#### 3.3.1. miRNA

**Figure 4:**
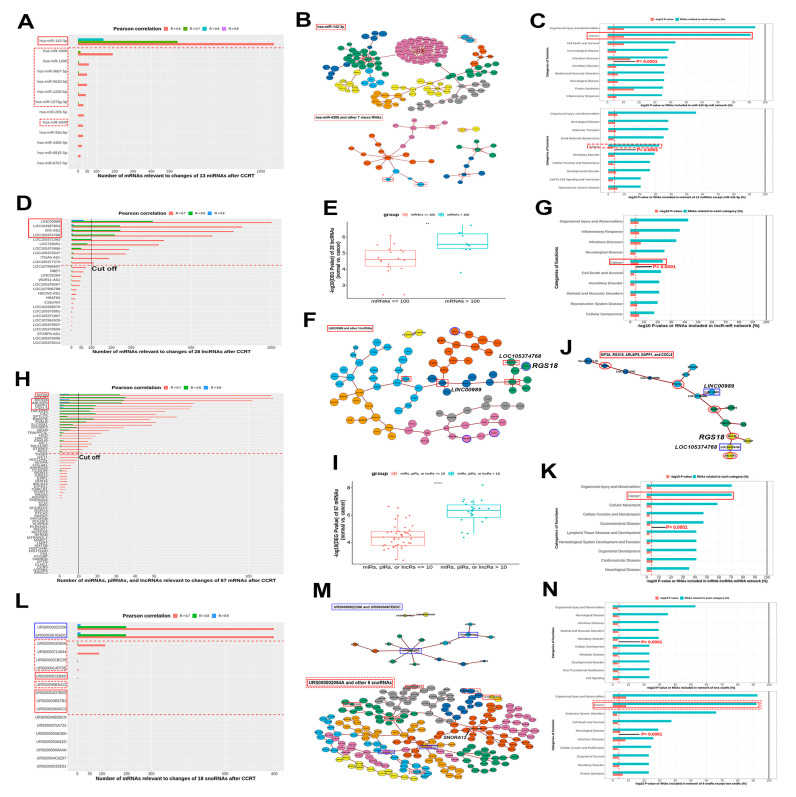
Biological screening of miRNAs, lncRNAs, mRNAs, and snoRNAs associated with cervical cancer. (**A**) A bar chart of the number and Pearson’s correlation of mRNAs relative to the initial 13 miRNAs. (**B**) A network of 139 mRNAs relative to miR-142-3p and the 13 miRNAs (upper), and a network of 28 mRNAs relative to other eight miRNAs and 13 miRNAs (lower). (**C**) The top 10 categories by relevance are sorted by percentage of RNAs relative to each category in a network with miR-142-3p (upper) and eight other miRNAs (lower). (**D**) A bar chart of the number and Pearson’s correlation of mRNAs relative to the 28 initially selected lncRNAs. (**E**) A comparison of −log_10_(DEG *p*-values) between lncRNAs with related mRNAs > 100 and those with related mRNAs ≤ 100. (**F**) A network of 76 mRNAs whose expression is affected by four lncRNAs with R > 0.9 and 28 lncRNAs. (**G**) The top 10 categories by relevance are sorted by percentage of RNAs relative to each category in the lncRNA-mRNA network. (**H**) A bar chart of the number and Pearson’s correlation of miRNAs, piRNAs, and lncRNAs relative to the initial 67 selected mRNAs. (**I**) A comparison of −log_10_(DEG *p*-values) between mRNAs with related ncRNAs > 10 and ncRNAs ≤ 10. (**J**) A network of six ncRNAs whose expression is altered by five mRNAs with R > 0.9 and the 68 mRNAs. (**K**) The top 10 categories by relevance are sorted by percentage of RNAs relative to each category in the mRNA-ncRNA network. (**L**) A bar chart of the number and Pearson’s correlation of mRNAs relative to the initial 18 selected snoRNAs. (**M**) A network of 13 mRNAs affected by URS0000822206 and URS000067E6DC (upper), and a network of 207 mRNAs relative to nine other snoRNAs (lower). (**N**) The top 10 categories by relevance are sorted by percentage of RNAs relative to each category in the networks of two (upper) and nine snoRNAs (lower).

The number and Pearson’s correlation of mRNAs associated with the log_2_FCs of 13 miRNAs identified after CCRT are presented in Figure 4A. There were significantly more mRNAs whose expression was altered by hsa-miR-142-3p than the other 12 miRNAs studied. A network with 139 mRNAs affected by hsa-miR-142-3p (R > 0.8) was more relative to the cancer category than the 28 mRNAs that were affected by has-miR-4306 and the other seven miRNAs (R > 0.7). The log_2_ (RPM+1) values of hsa-miR-142-3p were significantly lower in the cancer group than in the normal group regardless of pathology, while there was no significant change according to disease stage (Supplementary Figure S3A).

#### 3.3.2. lncRNA

The number and Pearson’s correlation of mRNAs associated with log_2_FCs of 28 lncRNAs after CCRT were sorted in descending order (Figure 4D). A cut-off value of 100 was used to maximize the −log_10_(DEG *p*-value) between the two groups of 28 lncRNAs according to the number of related mRNAs (R > 0.7; Supplementary Figure S2A). A group of lncRNAs with related mRNAs > 100 had a significantly higher −log_10_(DEG *p*-value) than the group with related mRNAs ≤ 100 (Figure 4E). This meant that the number of mRNAs relative to lncRNAs expressed after CCRT was positively correlated with the degree of statistical significance between the normal and cancer groups. Four lncRNAs with related mRNAs > 100 were selected owing to the presence of mRNAs with R > 0.9. A network indicated 76 mRNAs altered by LINC0089 and the other three lncRNAs that were relative to the cancer category (Figure 4F,G). The log2 (RPKM+1) values for these four lncRNAs were significantly lower in the cancer group than in the normal group regardless of pathology, while there was no significant change according to disease stage, except for LOC105374768 (Supplementary Figure S3B).

#### 3.3.3. mRNA

The number and Pearson’s correlation of miRNAs, piRNAs, and lncRNAs associated with the log_2_FCs of 67 mRNAs expressed after CCRT were sorted in descending order (Figure 4H). A cut-off value of 10 was used to maximize the −log_10_(DEG *p*-value) between two groups of 67 mRNAs according to the number of related ncRNAs (R > 0.7; Supplementary Figure S2B). A group of mRNAs with related ncRNAs > 10 had a significantly higher −log_10_(DEG *p*-value) than the group of mRNAs with related ncRNAs ≤ 10 (Figure 4I). This meant that the number of ncRNAs that were relative to the mRNAs expressed after CCRT was positively related to statistical significance between the normal and cancer groups. Five mRNAs with related ncRNAs > 10 were selected because of the presence of ncRNAs with R > 0.9. A network constructed from five lncRNAs and one miRNA that were affected by five mRNAs showed a significant association with the cancer category (Figure 4J,K). The log2 (RPKM+1) values of five mRNAs were significantly lower in the cancer group than in the normal group regardless of pathology, while there was no significant change according to disease stage, except for *CXCL5* (Supplementary Figure S3C).

#### 3.3.4. snoRNA

The number and Pearson’s correlation of mRNAs associated with log_2_FCs for 18 snoRNAs after CCRT are presented in Figure 4L. There were significantly more mRNAs affected by URS0000822206 or URS000067E6DC than by the other nine snoRNAs. A network of 207 mRNAs affected by URS000002084A and eight other snoRNAs (R > 0.7) was closely related to the cancer category, while a network of 13 mRNAs that were changed by URS0000822206 and URS000067E6DC (R > 0.9) was not (Figure 4M,N). The log2 (RPM+1) values of four snoRNAs that were not included in the ***DEG in snoRNA*** group were significantly higher in the cancer group than in the normal group regardless of pathology, while there was no significant change according to disease stage. (Supplementary Figure S3D).

#### 3.3.5. piRNA, snRNA, tRNA, and yRNA

The initial selection process of ncRNAs was similar to that of the lncRNAs for four classes of piRNAs, snRNAs, tRNAs, and yRNAs (Supplementary Figures S1, S2C–F and S3E–H).

### 3.4. DEG in snoRNA

**Figure 5:**
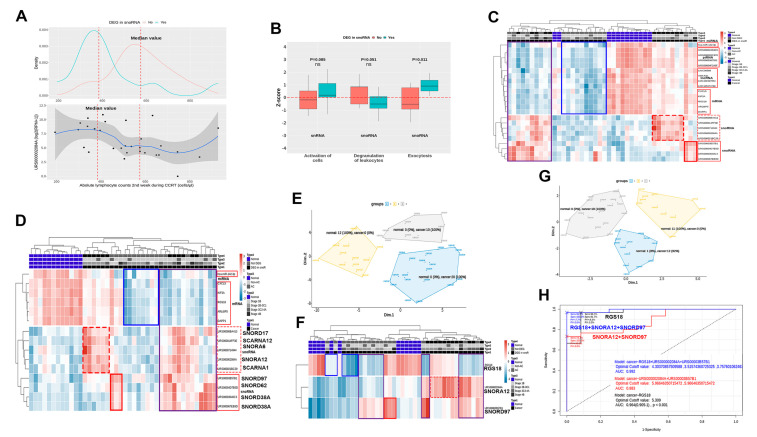
(**A**) Association between the log_2_(RPM+1) value of SNORA12 and the absolute lymphocyte counts in the second week of concurrent chemoradiotherapy according to the DEGs in snoRNA. (**B**) Subcategories that show the difference in Z-scores according to the DEG, in snoRNA in snoRNA or snoRNA. (**C**) An integrated heatmap of one miRNA, four piRNAs, four lncRNAs, five mRNAs, and nine snoRNAs that were selected through the screening process. (**D**) A heatmap and (**E**) a clustered multidimensional scaling (MDS) scatter plot of the combination of one miRNA, five mRNAs, and nine snoRNAs that can distinguish between the normal and cancer groups. (**F**) A heatmap and (**G**) a clustered multidimensional scaling (MDS) scatter plot of the combination of *RGS18*, SNORA12, and SNORD97, which can distinguish between the normal and cancer groups. (**H**) Three receiver operative characteristics curves of *RGS18* (black), *RGS18*+SNORA12+SNORD95 (blue), and SNORA12+SNORD95 (red).

The 12 patients with ***DEG in snoRNA*** had significantly lower ALC2 levels and higher pretreatment tumor marker levels than the other 18 patients (Table 1). Of the five snoRNAs included in the ***DEG in snoRNA*** group (Figure 4L, red dotted line), the log_2_ (RPM+1) values of URS000002084A (*SNORA12*) were most relative to ALC2 according to ***DEG in snoRNA*** (R^2^ = 0.19; Figure 5A). The difference in Z-score according to ***DEG in snoRNA*** was interpreted as the secondary reward activation (activation of cells↑, exocytosis↑) following weakened anticancer activity (degranulation of leukocytes↓; Figure 5B).

### 3.5. Integration

The snRNAs, tRNAs, and yRNAs were excluded from integration owing to their low sensitivity in the MDS plots (Figure 3E,H,K); however, five of the selected nine snoRNAs were DEGs between the two groups based on the ***DEG in snoRNA*** (Figure 5C,D, red dotted line). First, the miRNAs, piRNAs, lncRNAs, mRNAs, and snoRNAs selected via the two screening process steps were visualized as a hierarchical clustering heatmap (Figure 5C). This heatmap was divided into three groups, a group of 10 patients with increased snoRNAs (two red lines), a group of 10 patients with decreased miRNA, piRNAs, lncRNAs, and mRNAs (blue line), and a group of 10 patients with both increased snoRNAs and four classes of decreased RNAs (purple line). Second, we selected a combination of miRNAs, mRNAs, and snoRNAs to distinguish the normal and cancer groups after MDS and hierarchical clustering heatmap analyses with various combinations that included snoRNAs (Figure 5D,E). Third, we selected *RGS18*, SNORA12, and URS00003B57B1 (SNORD97), which were visualized as an MDS plot and a hierarchical clustering heatmap (Figure 5F,G). In mRNA and lncRNA networks, *RGS18* was interrelated with LOC105374768 and LINC00989, which were selected through the lncRNA screening process (Figure 4F,J). We selected SNORA12, which was at the center of the snoRNA network (Figure 4M, lower) and closely related to ALC2 (Figure 5A). Excluding the five snoRNAs in the ***DEG in snoRNA*** group, the snoRNA combinations that included SNORD97 were better, than those that included the other three snoRNAs, at distinguishing between the normal and cancer groups in the MDS or heatmap analyses. The ROC curves for cancer diagnosis using log_2_(RPM+1) or log_2_(RPKM+1) showed that *RGS18*, SNORA12, and SNORD97 had higher area under the curve (AUC), sensitivity, and specificity than *RGS18* alone or both SNORA12 and SNORD97 (0.992, 96.7%, and 100% for *RGS18*, SNORA12, and SNORD97; 0.964, 96.7%, and 91.7% for *RGS18*; and 0.883, 73.3%, and 100% for SNORA12 and SNORD97; Figure 5H).

## 4. Discussion

To select plasma exosomal ncRNA (or mRNA) for the diagnosis of cervical cancer, we targeted RNAs that had statistical differences in expression levels between the normal and cancer patient groups. Candidate ncRNAs (or mRNAs) were selected based on the increase in the number of mRNAs (or ncRNAs) altered by log_2_FC of ncRNAs (or mRNAs) and the Pearson’s correlation between mRNAs and ncRNAs. The association between ncRNA-mRNA networks and cancer category was confirmed using IPA. The process for additionally exploring the changes in RNA expression in response to CCRT helped in the efficient selection of many RNAs with statistical significance from various classes. The core of this process was based on the hypothesis that the number of cancer cells can be reduced by irradiation, which alters the expression of cancer-associated RNA that has a stronger biological influence on the size and correlation of the ncRNA-mRNA network.

When the RNAs present in exosomes were selected and integrated for all classes, they were broadly divided into four classes of miRNAs, piRNAs, lncRNAs, and mRNAs and a single class of snoRNAs (Figure 5C). In particular, the combination of miRNA-mRNA-snoRNA clearly distinguished the normal group from the cancer group (Figure 5D,E). This can imply various characteristics, including growth, invasion, metastasis, neovascularization, evasion of tumor suppression, genetic instability, inflammation, immune evasion, and alteration of energy metabolism, which are acquired by the tumor microenvironment (TME), as shown in Table 2 [19].

Previous studies have suggested that expression of miR-142-3p, which suppresses tumor proliferation and invasion, is not only reduced in cervical cancer tissues, compared with that in adjacent normal cervical tissue, but is also associated with prognosis [20,21]. Cunha et al. [22] reported that *ARL6IP5*, which was one of the selected five mRNAs and included in the tumor suppressor gene database (https://bioinfo.uth.edu/TSGene/, accessed on 1 September 2021), was downregulated more in the soft tissue sarcoma tissues than in benign tumors. The reduced expression of miR-142-3p and *ARL6IP5* in plasma exosomes from the cancer group may indicate their role as tumor suppressors during cervical tumorigenesis. The expression of *CXCL5* and *KIF2A* was upregulated in various cancer tissues, including cervical cancer [24,25,27,38]; additionally, the expression of *RGS18* in the tissues of patients with ovarian cancer was also upregulated [30]. However, each of the three mRNAs can contribute to tumorigenesis through a different biological mechanism. CXCL5 from tumor cells can promote tumor growth and angiogenesis by recruiting tumor and immune cells into the TME [23]. We might suggest a negative correlation between CXCL5 and cervical cancer stage if CXCL5 from blood plasma exosomes is absorbed and secreted into the TME by tumor cells. The expression level of KIF2A in exosomes could be lower in the cancer group than in the normal group if tumors absorbed *KIF2A* required for cell mitosis and proliferation [26]. *RGS18* deficiency can reportedly lead to platelet activation and reduce platelet survival, whereas its abundance can inhibit platelet activation and promote platelet production [28,29]. The decrease in plasma exosomal *RGS18* in the cancer group may be attributed to the absorption of *RGS18* by cervical cancer cells. This may result in the promotion of tumorigenesis and tumor progression by facilitating tumor-associated platelet activation, and the presence of *RGS18* in the tumor may contribute to sustained blood platelet concentration through its transfer to hematopoietic stem cells. According to a preclinical study, *DAPP1* deficiency could not efficiently activate antigen-specific T cells [31], suggesting that *DAPP1* downregulation is associated with a weakness in cancer-specific immunity. Four mRNAs (*KIF2A*, *RGS18*, *ARL6IP5*, and *DAPP1*) and two lncRNAs (LINC00989 and LOC105374768) were included in both the mRNA-ncRNA network and lncRNA-mRNA network (Figure 4F,J). This study showed that the log_2_FC of *RGS18* with CCRT strongly correlated with two lncRNAs in the network. A previous study on the RNA expression of tumor-educated platelets (TEPs) revealed that the expression level of both *RGS18* and LINC00989 was lower in platelets from cancer patients than in those from healthy donors [32]. This supports that the two lncRNAs may be associated with tumorigenesis through RGS18-mediated platelet activation. In addition, TEPs may secrete *CXCL5*, which can promote the recruitment of leukocytes into the TME [39]. Therefore, plasma exosomal *RGS18* may be more associated with cervical cancer development and progression than the other three mRNAs (*KIF2A*, *ARL6IP5*, and *DAPP1*).

The nine selected snoRNAs were different from other classes of selected RNAs in that the number of mRNAs relative to the log_2_FC of the snoRNAs was smaller than that of the mRNAs relative to the change in other selected RNAs following CCRT (Figure 4 and Supplementary Figure S1). Therefore, the two snoRNAs with many relative mRNAs (Figure 4L) may function as a normal physiological response following CCRT. Unlike other ncRNAs, snoRNA contained seven RNAs that were specifically upregulated in 12 patients with cancer ***(DEG in snoRNA***), and many of their snRNA, tRNA, and yRNA genes were differentially expressed in the other 30 patients (Figure 3). These RNAs were associated with both a decrease in ALC2 levels and an increase in pretreatment tumor marker expression (Table 1) and may contribute to a weakening of immunity that was associated with cancer according to the IPA (Figure 5B). Previous studies of the changes in snoRNA content in extracellular vesicles of immune cells using immunosuppression or immunostimulatory factors and snoRNA secretions stimulated by inflammation suggested that snoRNA functions as a modulator of inflammation [40,41,42]. In addition, RNAs further processed from snoRNAs, including SNORD17 or SNORA6, among seven snoRNAs ***(DEG in snoRNA***), were associated with the number of tumor-infiltrating CD8 T cells [33]. Therefore, we can assume that cervical cancer may be associated with snoRNA, which would reflect an impaired inflammatory or immune response followed by lymphopenia. Additionally, lymphopenia in patients with cervical cancer was associated with clinical results of CCRT [43,44], indicating that ***DEG in snoRNA*** may be related to cervical cancer prognosis. We selected SNORA12 as the most relative to ALC2 among the five selected ***DEGs in snoRNA*** (Figure 5A,D, red dotted line), which showed that snoRNA was more suitable for diagnosing cancer than the other three RNA classes (e.g., snRNA, tRNA, and yRNA) because it had an additional four RNAs that were differentially expressed in the cancer group (Figure 5D, red solid line). snoRNAs may also be relevant to cancer immunity, considering the association between SNORD38A (URS00003640C3 or URS000067EB9D) of the four snoRNAs and the number of tumor-infiltrating CD8 T cells [33]. Among the four selected snoRNAs, the combination of SNORD97 with both *RGS18* and SNORA12 was the most appropriate for distinguishing between the normal and cancer groups using the MDS plot, heatmap, and ROC curve analyses (Figure 5F,G,H). The downregulation of SNORA12 and SNORD97 in tissues from patients with cervical squamous cell carcinoma [37] implies that their secretion from cancer cells may help to evade host immunity against tumors through suppression of both systemic lymphopenia and lymphocyte activity in the TME. Taken together, our data suggest that the 15 plasma exosomal RNAs from the three classes of miRNA, mRNAs, and snoRNAs distinguish the normal and cancer groups by reflecting the evasion of tumor suppression (miR-142-3p and *ARL6IP5),* tumor proliferation (*KIF2A*), tumor progression through the TME (*CXCL5* and *RGS18*), and cancer immunity (*DAPP1* and 9 snoRNAs). In particular, tumor-related platelet activation by downregulation of *RGS18* and an immune suppression by upregulation of both SNORA12 and SNORD97 may be essential mechanisms for cervical cancer development and progression.

We report a secondary screening method using the log_2_FC of mRNAs (or ncRNAs) before and during CCRT in patients with cervical cancer and the potential diagnostic RNAs selected by this method; however, there are several limitations to these results and require further investigation. First, biases can arise from the process of obtaining and analyzing RNA sequences, such as the heterogeneity of exosomes and their RNAs due to CCRT, the different methods used to isolate exosomes, and the different RNA profiling methods [42]. Therefore, the reproducibility of the present study is not guaranteed. Second, this is a preliminary analysis with a small number of patients, and therefore, the reliability of this study is limited. Third, additional validation of RNAs selected using the proposed screening method is still required, including biological mechanism characterization or animal studies for the selected RNAs presented in this study. We attempted to overcome the bias of the RNA sequencing analytic process by performing paired comparisons of the 60 samples from 30 patients before and during CCRT. Moreover, the 30 samples collected during CCRT were irradiated at a constant dosage per time point (approximately 18 Gy every 2 weeks) and cisplatin regimen (30–70 mg/m^2^ every week), as reported in a previous study [17]. However, the reliability of the reported methodology should be validated by applying a similar method to select plasma exosomal RNAs for diagnosis using various sizes of datasets from different cancer patients treated with RT. Furthermore, the 15 RNAs selected from this pilot study should be verified in a large cohort dataset and validated with preclinical studies, despite their diagnostic potential for cervical cancer and implications as hallmarks of cancer.

## 5. Conclusions

We present the first method for efficiently screening cancer-related RNAs using mRNAs (or ncRNAs) relative to the log2FC of ncRNAs (or mRNAs) altered by CCRT and suggest the association between the RNAs identified by this method and their dysregulation in cancer. This method may reduce the time and resources needed to develop diagnostic and therapeutic biomarkers for cervical cancer and should be validated in further preclinical and clinical studies.

## Figures and Tables

**Table 1:** Patient clinical characteristics.

	Normal	Cancer	*DEG in snoRNA*		*p*
	(*n* = 12)	(*n* = 30)	No (*n* = 18)	Yes (*n* = 12)	
Age (years)	49.2 ± 11.6	49.9 ± 10.1	47.9 ± 11.4	52.8 ± 7.1	0.199
FIGO stage 2018					0.627
- IB		5 (16.7%)	4 (22.2%)	1 (8.3%)	
- IIB-IIIC1		15 (50.0%)	9 (50.0%)	6 (50.0%)	
- IIIC2-IVA		7 (23.3%)	4 (22.2%)	3 (25.0%)	
- IVB		3 (10.0%)	1 (5.6%)	2 (16.7%)	
Pathology					0.374
- Adenocarcinoma		5 (16.7%)	4 (22.2%)	1 (8.3%)	
- Adenosquamous cell carcinoma		1 (3.3%)	0 (0.0%)	1 (8.3%)	
- Unclassified carcinoma		1 (3.3%)	1 (5.6%)	0 (0.0%)	
- Squamous cell carcinoma		23 (76.7%)	13 (72.2%)	10 (83.3%)	
Radiotherapy field					0.464
Pelvis		21 (70.0%)	14 (77.8%)	7 (58.3%)	
Pelvis with paraaortic region		9 (30.0%)	4 (22.2%)	5 (41.7%)	
Hemoglobin (g/dl)					
Pretreatment		12.1 ± 1.5	12.0 ± 1.5	12.2 ± 1.6	0.78
Second week during CCRT		11.1 ± 1.4	11.3 ± 1.2	10.8 ± 1.7	0.336
Absolute lymphocyte count (cells/μL)					
Pretreatment		1754 ± 470	1758 ± 451	1747 ± 518	0.95
First week after CCRT		931 ± 393	929 ± 281	936 ± 546	0.966
Second week after CCRT		511 [371; 632]	575 [505; 661]	384 [323; 466]	0.008
Pretreatment tumor marker (ng/mL)					
Squamous cell carcinoma antigen		3.7 [0.9; 16.6]	2.2 [0.8; 4.8]	13.1 [4.0; 60.6]	0.016
Cytokeratin fragment 21-1		2.5 [1.8; 10.2]	2.2 [1.2; 2.8]	8.4 [2.5; 16.6]	0.031
Pretreatment tumor volume (cm^3^)		50.5 [18.1; 94.1]	40.6 [15.2; 94.1]	61.0 [30.9; 103.3]	0.346

FIGO—International Federation of Gynecology and Obstetrics; CCRT—concurrent chemoradiotherapy; DEG—differentially expressed genes; snoRNA—small nucleolar RNA. Continuous variables described using median [interquartile range] or mean ± standard deviation.

**Table 2:** Suggested biological functions of selected plasma exosomal RNAs.

RNA	Known Biological Functions	Tissue	Suggested Biological Functions	Exosome
miR-142-3p	Tumor suppressor [20]	↓(CC) [21]	Tumor suppressor	↓
ARL6IP5	Tumor suppressor (https://bioinfo.uth.edu/TSGene/ accessed on 1 September 2021)	↓(STT) [22]	Tumor suppressor	↓
CXCL5	Recruits and activates granulocytes and promotes angiogenesis, tumor growth, and metastasis in the tumor microenvironment [23]	↑(CC) [24,25]	Tumors with exosome-derived CXCL5 use it to facilitate their progression through infiltration of leukocytes in the tumor microenvironment	↓
KIF2A	Required for cell mitosis [26]	↑(CC) [27]	Rapid mitosis of cancer cells may promote the absorption of *KIF2A* from exosomes	↓
RGS18	Negative regulator of G protein-coupled receptors and controls platelet activation and production [28,29]	↑(OC) [30]	Tumors may absorb *RGS18* present in exosomes, which can promote thrombogenesis. The reduction of exosomal *RGS18* by tumors may promote activated platelets around the primary tumor, which can facilitate tumor growth and invasion. Therefore, dysregulation of *RGS18* can result in tumorigenesis through persistent platelet activation	↓
DAPP1	Activation of antigen-specific T cells [31]	NA	This may contribute to tumorigenesis through deficiency of tumor-specific immunity	↓
LINC00989	Decreases with RGS18 in tumor-educated platelets [32]	↓(PaC) [32]	The two lncRNAs may facilitate platelet activation in cancer patients via targeting *RGS18*	↓
LOC105374768	NA	NA		↓
SNORD17	The derived RNA positively correlates with CD8 T cell infiltration in thymoma and stomach cancer [33]	↑(COC) [34]	Promotion of these snoRNAs present in exosomes may be related to cancer related-lymphopenia	↑
SCARNA12	NA	↑(LC) [35]		↑
SNORA6	The derived RNA negatively correlates with CD8 T cell infiltration in LGG, PC, pancreatic cancer, and HNC [33]	↑(PC) [36]		↑
SNORA12	NA	↓(CC) [37] ↑(LC) [35]		↑
SCARNA1	NA	↑(LC) [35]		↑
SNORD97	NA	↓(CC) [37]	Promotion of these snoRNAs present in exosomes may be related to decreased lymphocyte activity	↑
SNORD62	NA	NA		↑
SNORD38A	The derived RNA negatively correlates with CD8 T cell infiltration in HNC, LC, TGCT, and PCPG [33]	↑(COC) [34]		↑

CC—cervical cancer; STT—soft tissue tumor; OC—ovarian cancer; PaC—Pan cancer; COC—colon cancer; LC—lung cancer; LGG—low grade glioma; PC—prostate cancer; HNC—head and neck cancer; TGCT—testicular germ cell tumor; PCPG—pheochromocytomas and paragangliomas; SNORD38A includes URS00003640C3 or URS000067EB9D.

## Data Availability

All data analyzed during this study are available. Original sequencing data: ArrayExpress (accession number: E-MTAB-10215, E-MTAB-10930) Coding and dataset: https://data.mendeley.com/datasets/bnzzvk38d8/draft?a=f5ea1853-dcf8-4f74-ba61-a8e527a11a9e.

## Supplementary Materials

The following are available online at https://www.mdpi.com/article/10.3390/biom11111691/s1, Supplementary Method; Supplementary Figures S1–S3; Supplementary Figure legend.
