# Supplementary material for: Screening Plasma Exosomal RNAs as Diagnostic Markers for Cervical Cancer: An Analysis of Patients Who Underwent Primary Chemoradiotherapy
Source: Biomolecules. 2021 Nov 14;11(11):1691. doi: 10.3390/biom11111691 (PMC8615616; doi:10.3390/biom11111691)
Supplement: Supplementary file 1 [file biomolecules-11-01691-s001.zip › exosome isolation protocols using Exo2D.pdf]

EXOSOME plus

Exo2D^{TM} - for EV isolation

(Urine, Culture media, Saliva, Ascites fluid)

User manual

Store kit at +25 C on receipt
A limited-use label license covers this product. By use of this product, you accept the terms and conditions outlined in the Licensing and Warranty Statement contained in this user manual.

Contents

I. Overview 2
II. Principle 3
III. Protocol 4
IV. Sample data and applications 7

List of components

Item	Volume
EXO2D™ - L solution A	15 ml

Exo2D™ are shipped at room temperature and should be stored at +25°C upon receipt. Properly stored kits are stable for 6 month from the date received.
Exo2D™ can be used for urine, saliva, spinal cord, ascites, culture media, and etc.
Range of bio fluid volume is 50 ul to 100 ml.

Exo2D™ – for RNA analysis

I. Overview

Exosomes, small membrane vesicles secreted by cell, are found in blood, urine, amniotic fluid, malignant ascite fluids and contain distinct subsets of microRNAs and proteins. Although exosome have increasingly received attention by the field of diagnosis, regeneration, delivery, and etc., existing isolation methods including commercialized kit did not meet the users' needs because of low isolation efficiency and poor purity. To solve the unmet needs, we developed a new exosome isolation kit which mechanism is different from the previous ones. We guarantees almost perfect exosome isolation efficiency and highest purity. This may make impossible assays to possible assays.

- No time-consuming ultracentrifugation
- No incubation time
- Total process time is less than 1 h
- Almost perfect exosome isolation efficiency
- Purity of exosome can be improved by additional process
- High ability of RNA and miRNA PCR
- High ability of NGS

II. Principle

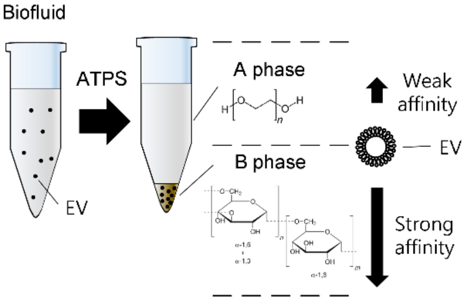

We developed a new exosome isolation methods, which is different from the conventional methods.

Following is a main mechanism of the conventional methods.

Ultracentrifugation: Use high g-force
Polymeric precipitation: Aggregate the exosome
Magnetic bead: Antibody-antigen reaction
Chromatography: Size exclusion or antibody-antigen reaction

Unlike the methods, we use surface property of exosomes.

We make an aqueous two phase, which is similar to oil and water phase.

Using the interactions between surface of exosome and each phase, we almost perfectly isolate exosome to one of phases.

III. PROTOCOL

Material delivered

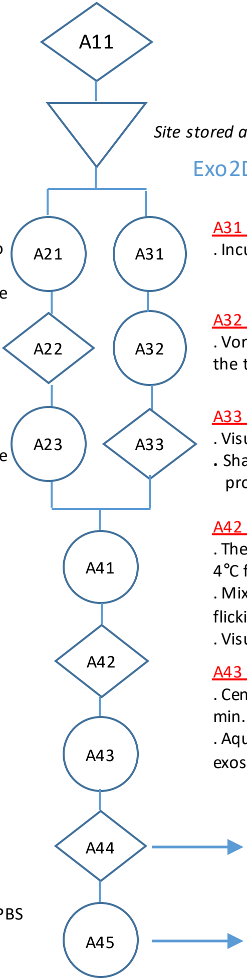

Site stored at 25°C

Urine, Culture media,
Saliva, Ascites fluid

Exo2D™-L Reagent A

A11 Incoming Inspection
. Check the labels
. Visual check : Damage, impurity, leakage during delivery

A21 Pre-cleaning
. Centrifuge at 3000×*g* for 15 min to remove cells and cell debris.
. Transfer supernatant to a new tube

A22 Inspection
. Visual check : **pellet**

A23 Sample collection
. Transfer supernatant to a new tube

A31 Incubation
. Incubate at 37°C for 15 min.

A32 Homogenization
. Vortexing, Inverting or flicking the tube

A33 Inspection
. Visual check : Evenly **OPAQUE**
. Shake every 5 min. during the process to keep optically opaque

A41 Injection & Mixing
. Immediately, add to 10 mℓ of sample per 2 mℓ of Exo2D™– L reagent A
. Mixing well by inverting or flicking

A42 Inspection
. The solution was incubated at 4°C for 30 min.
. Mixing well by inverting or flicking
. Visual check : Evenly **OPAQUE**

A43 Precipitation
. Centrifuge at 3,000 × g for 30 min. at 4°C.
. Aqueous phase of Exo2D™ grabs exosomes and precipitated

A44 Removal of supernatant
. Exosomes are dissolved in very small volume of aqueous phase of Exo2D™
. The phase appears as white pellets. The remainder should be eliminated.

Optional
Go to step B in case you need highly purified exosome

A45 Resuspending
. Resuspend bottom solution using PBS

Optional
Go to step C in case you use trizol based RNA isolation

III. PROTOCOL

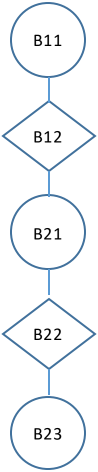

B11 Purification

. Add 5 ㎖ purification solution
. Mix well by inverting or flicking the tube

B12 Inspection

. Visual check : pellet was dissolved in purification solution

B21 Centrifugation

. Centrifuge the sample at 3,000 × g for 30 minutes at 4℃.
. Remove the supernatant.

B12 Inspection

. Visual check : pellet

A23 Resuspending

. Resuspend bottom solution using PBS

III. PROTOCOL

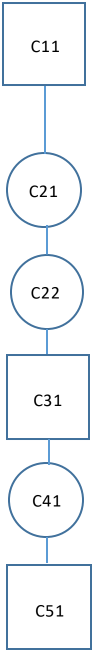

C11 Lysis and Phase sepration

You can use your protocol in this step

. add 1000 $\mu\ell$ of **Lysis Buffer** per 200 $\mu\ell$ of the isolated sample.
. Shake tube vigorously by vortex for 15 seconds.
. Incubate for 2 min at room temperature
. Add 300 $\mu\ell$ of chloroform per 1 mL of **Lysis Buffer.**
. Incubate for 2 min at 4℃
Shake tube vigorously by vortex for 15 seconds. . Centrifuge the sample at 12,000 × g for 15 min at 4℃.

C21 Purification

. Place the aqueous phase into a new tube, and add 1ml of chloroform.
. Shake the tube vigorously by vortex for 10 seconds.
. Place the sample on ice, and shake the sample for 30 min

C22 Sample extraction

. Centrifuge at 1000 × g for 5 minutes.
. Separate upper phase by pipette.

C31 RNA precipitation

You can use your protocol in this step

. Add same volume of isopropanol to the upper phase
. Incubate overnight at -80℃ or incubate at -20℃ for 30 min
. Centrifuge at 16,000 × g for 10 minutes at 4℃
. Remove the supernatant from the tube, leaving only the RNA pellet

C41 RNA purification

. Wash the pellet, with 1 mL of 70% ethanol (invert the tube) If there is white pellet in the bottom of tube, shake the tube by vortexing for 10 sec. The white material protects RNA and assists precipitation of RNA.
. Centrifuge at 16,000 × g for 10 minutes at 4℃
. Repeat step C41, after first purification, you don't need to vortexing the tube.

C51 Resuspending

You can use your protocol in this step

. Air dry the RNA pellet
. Resuspend the RNA pellet in 10 $\mu\ell$ RNase-free water

IV. Sample data and applications

A. Isolation efficiency of exosome

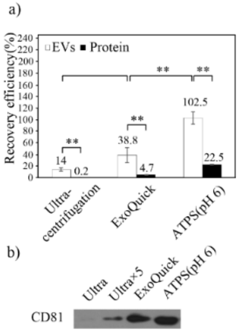

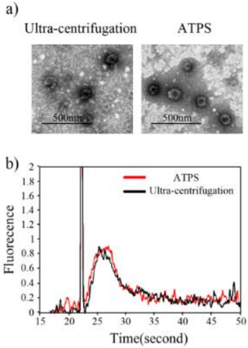

- Exosome isolation efficiency of ATPS is almost perfect.

B. Purity of exosome

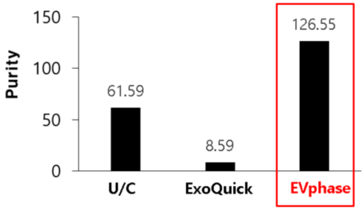

- Purity of isolated exosome by EVphase is highest among the methods.

IV. Sample data and applications

C. mi-RNA PCR

1. High miRNA performance – High purity

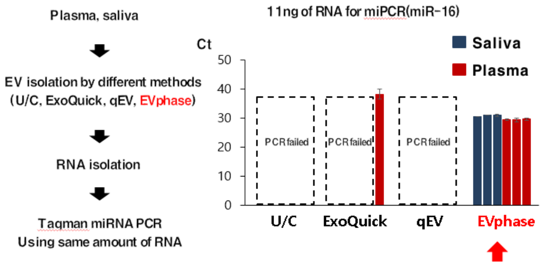

2. High miRNA performance – High yield

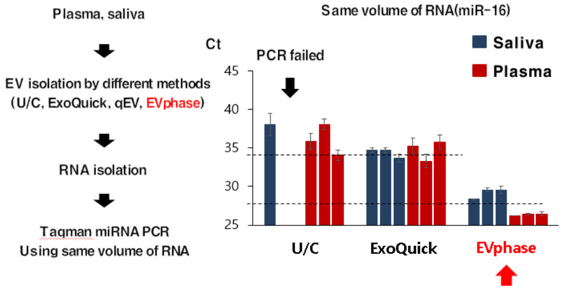

- Highest miRNA performance
- High reliability
