# Supplementary material for: Screening Plasma Exosomal RNAs as Diagnostic Markers for Cervical Cancer: An Analysis of Patients Who Underwent Primary Chemoradiotherapy
Source: Biomolecules. 2021 Nov 14;11(11):1691. doi: 10.3390/biom11111691 (PMC8615616; doi:10.3390/biom11111691)
Supplement: Supplementary file 1 [file biomolecules-11-01691-s001.zip › RNA extraction protocols using miRNeasy SerumPlasma Kit.pdf]

QIAGEN Serum/Plasma miRNA kit protocol

Material

.- QIAzol® Lysis Reagent (Cat. No. 79306), miRNeasy® Serum/Plasma Kit
- Store the RNeasy MinElute spin columns immediately at 2-8°C.

Method

Homogenizing Samples

1. Prepare serum of plasma, or thaw frozen samples.
2. Add 5 volumes Trizol to the sample. Mix by vortexing or pipetting up and down.
3. Incubate the homogenate at room temperature for 5min.

Phase separation

1. Add chloroform of an equal volume to the starting sample.(almost 280ul)
2. Mix by vortexting and Incubate at room temperature for 2-3minutes.
3. Centrifuge for 15 minutes at 12,000 x *g* at 4°C
4. Transfer the upper a queous phase to a 15ml conical tube.
5. Add 1.5 volumes absolute ethanol. Mix thoroughly by pipetting or inverting.

RNA wash & Elution

1. Transfer 700ul of each sample, including any precipitate that may have formed, to each RNeasy MinElute spin column on the vacuum manifold
2. Switch on the vacuum. Apply vacuum until transfer is complete. Switch off the vacuum and ventilate the vacuum manifold.
3. If necessary, repeat steps 1 and 2 with the remaining volume of each sample.
4. Add 700ul of RWT buffer to each RNeasy MinElute spin column.
5. Repeat step 2.
6. Add 500ul of RPE buffer to each RNeasy MinElute spin colum.
7. Repeat step 2.
8. Add 500ul of 80% ethanol to each RNeasy MinElute spin colum.
9. Repeat step 2.
10. Remove the RNeasy MinElute spin columns from the vacuum manifold, and place each in a 2 ml collection tube. Close the lids gently, and centrifuge at 12,000 x *g* for 30 seconds.
11 Place each RNeasy MinElute spin columns in a new 1.5ml tube. Add 20ul RNase-free water directly to each spin column membrane. Incubate for 3 minutes at room temperature.
12. Centrifuge for 1 minutes at full speed.
