# Supplementary material for: Screening Plasma Exosomal RNAs as Diagnostic Markers for Cervical Cancer: An Analysis of Patients Who Underwent Primary Chemoradiotherapy
Source: Biomolecules. 2021 Nov 14;11(11):1691. doi: 10.3390/biom11111691 (PMC8615616; doi:10.3390/biom11111691)
Supplement: Supplementary file 1 [file biomolecules-11-01691-s001.zip › Supplementary_figures and legends.pdf]

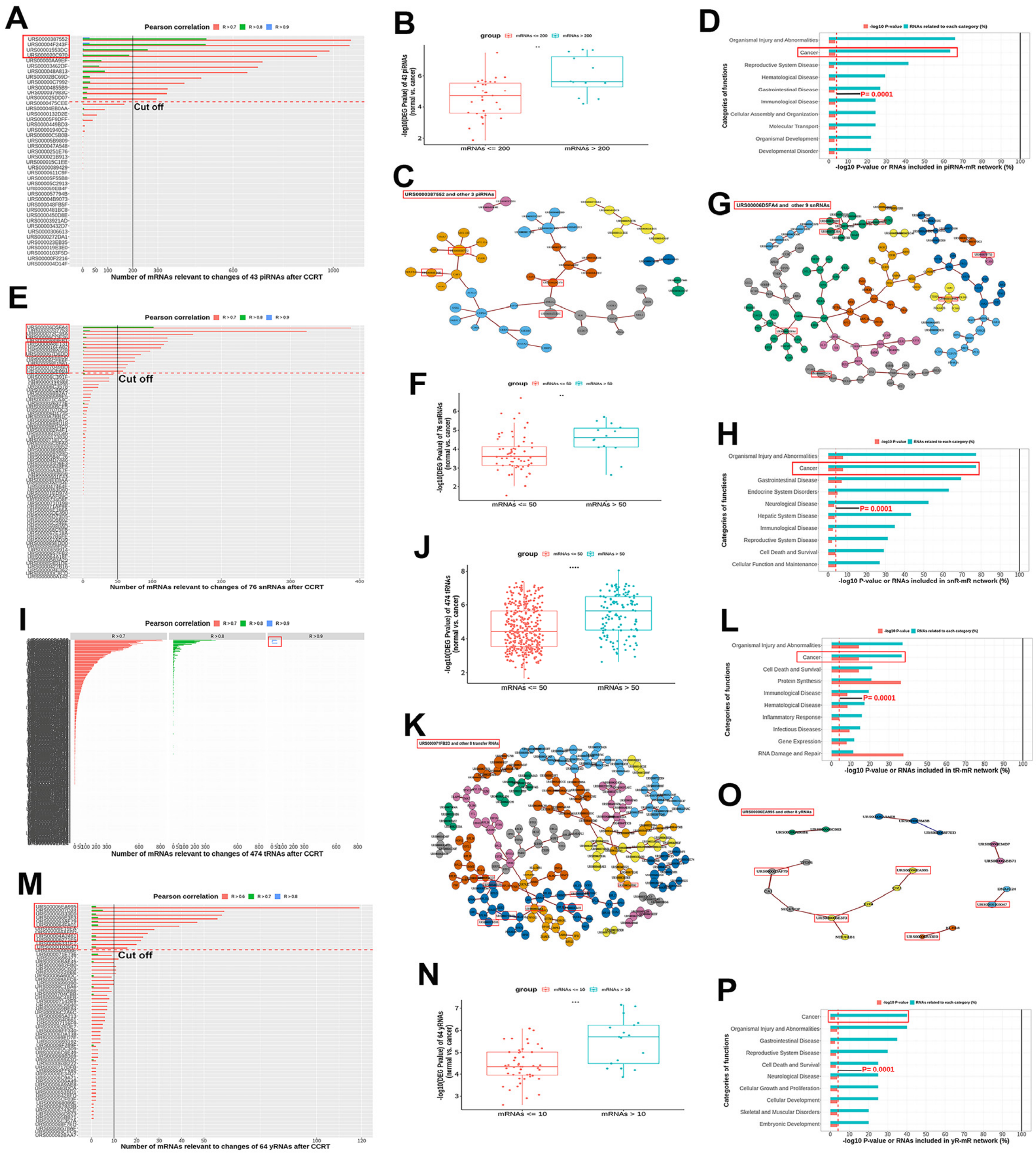

Supplementary Figure S1. Biological screening of piRNAs, snRNAs, tRNAs, and yRNAs isolated from plasma exosomes. (A) A bar chart of the number and Pearson's correlation of mRNAs relative to the 43 primarily selected piRNAs. (B) A comparison of -$\log_{10}$(DEG P values) between piRNAs with related mRNAs > 200 and those with related mRNAs ≤ 200. (C) A network of 28 mRNAs altered by four piRNAs with R > 0.9 and 43 lncRNAs. (D) The top 10 categories by relevance were sorted by percentage of RNAs relative to each category in the piRNA-mRNA network. (E) A bar chart of the number and Pearson's correlation of mRNAs relative to the 76 primarily selected snRNAs. (F) A comparison of -$\log_{10}$(DEG P values) between snRNAs with related mRNAs > 50 and those with related

mRNAs ≤ 50. (G) A network of 110 mRNAs affected by 10 snRNAs with R > 0.8 and 76 snRNAs. (H) The top 10 categories by relevance were sorted by percentage of RNAs relative to each category in the snRNA-mRNA network. (I) A bar chart of the number and Pearson's correlation of mRNAs relative to the 474 primarily selected tRNAs. (J) A comparison of $-\log_{10}$(DEG *P* values) between tRNAs with related mRNAs > 50 and those with related mRNAs ≤ 50. (K) A network of 88 mRNAs affected by 9 tRNAs with R > 0.9 and 474 tRNAs. (L) The top 10 categories by relevance were sorted by the percentage of RNAs relative to each category in the tRNA-mRNA network. (M) A bar chart of the number and Pearson's correlation of mRNAs relative to the 64 primarily selected yRNAs. (N) A comparison of $-\log_{10}$(DEG *P* values) between the yRNAs with related mRNAs > 10 and those with related mRNAs ≤ 10. (O) A network of eight mRNAs affected by 9 yRNAs with R > 0.7 and 64 yRNAs. (P) The top 10 categories by relevance were sorted by percentage of RNAs relative to each category in the yRNA-mRNA network.

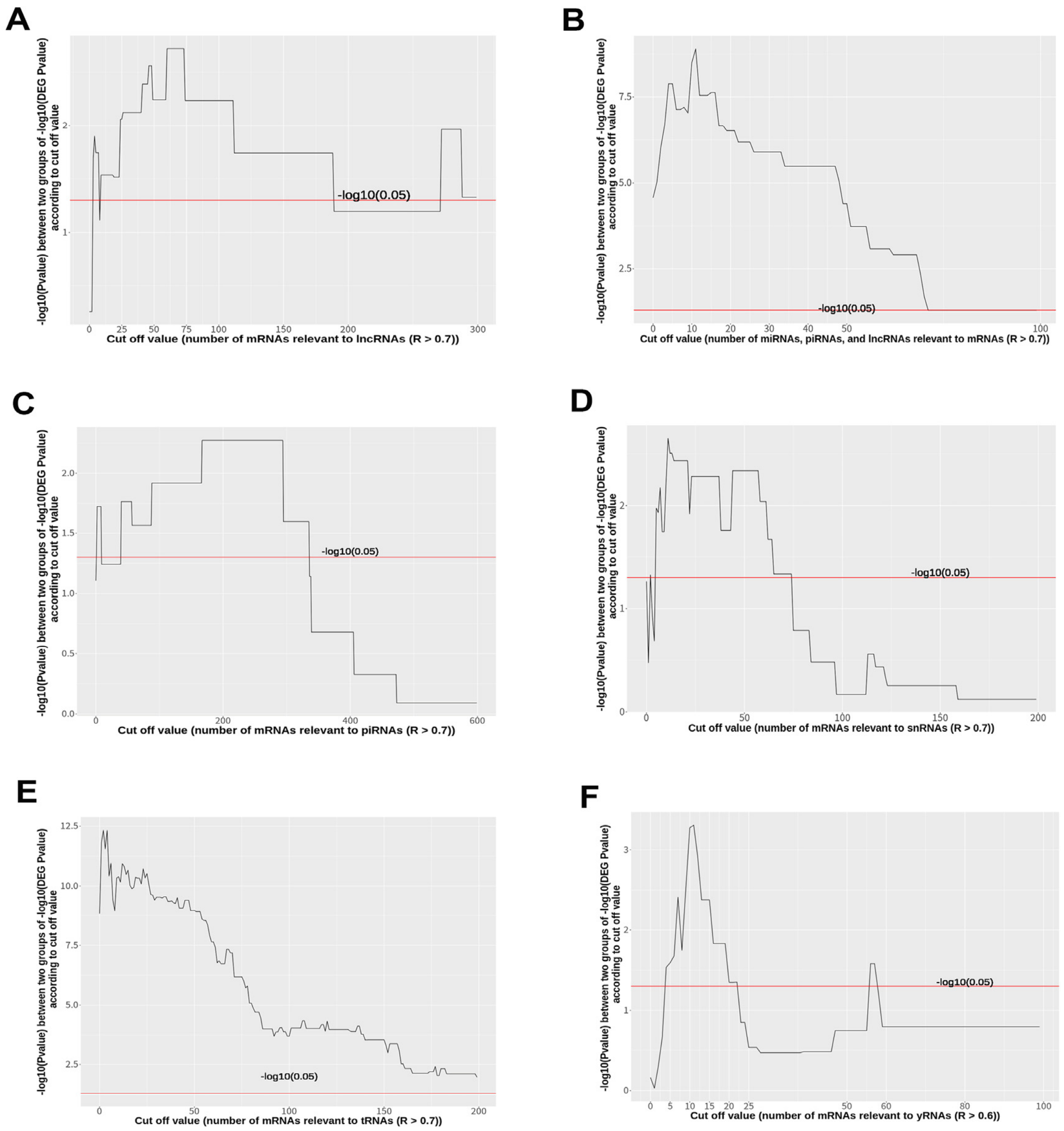

Supplementary Figure S2. A comparison between two groups of -log10(DEG *P* value) in ncRNA (or mRNA) according to the cut off value, and the number of mRNAs (or ncRNAs) relative to ncRNAs (or mRNAs; R > 0.7). The graph was used to determine the cutoff values for (A) lncRNAs, (B) mRNAs, (C) piRNAs, (D) snRNAs, (E) tRNAs, or (F) yRNAs isolated from plasma exosomes.

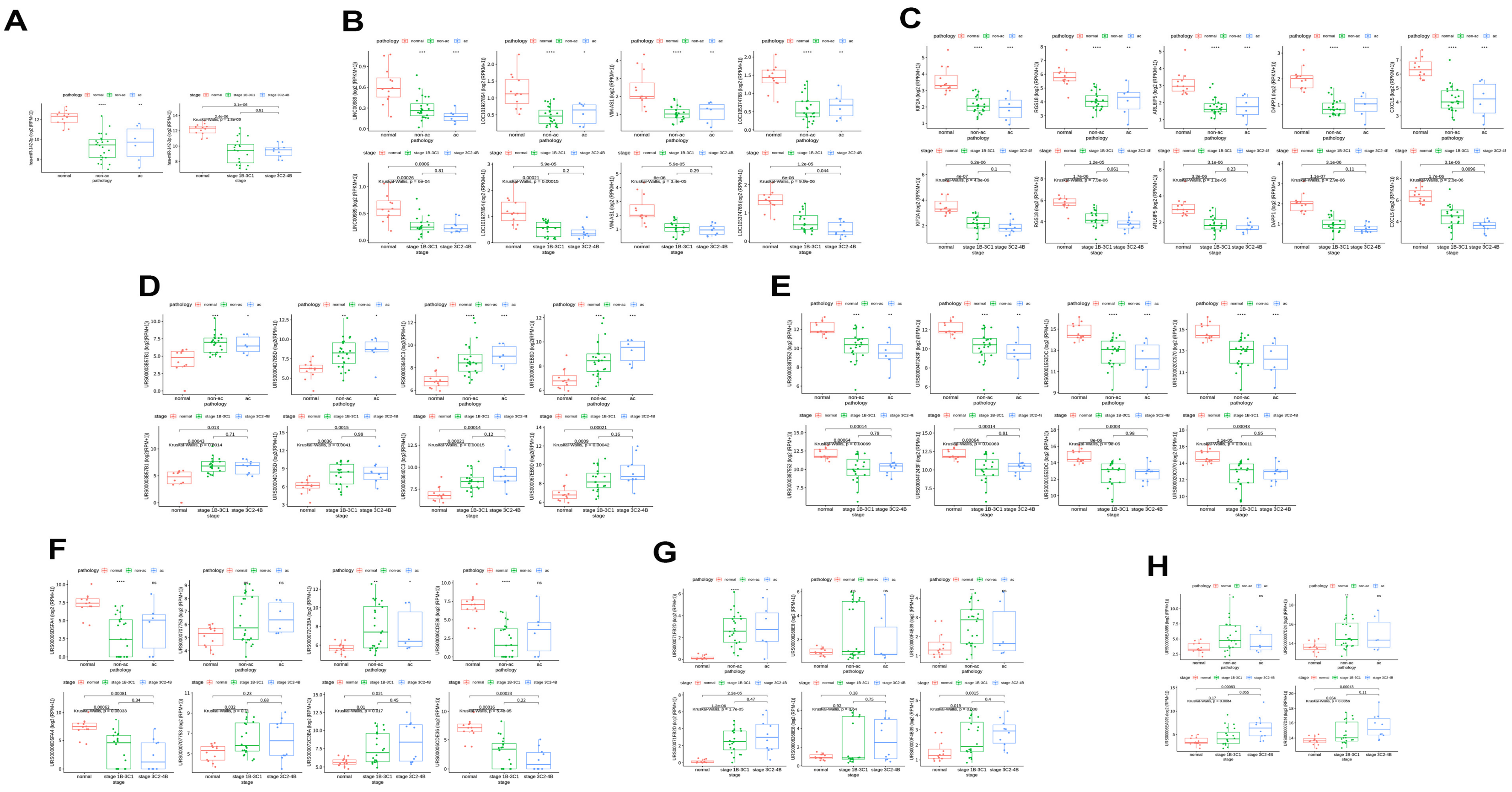

Supplementary Figure S3. Boxplots comparing the $\log_2(RPM+1)$ or $\log_2(RPKM+1)$ values among the normal, non-ac (squamous cell or unclassified carcinoma), and ac (adeno or adnosquamous cell carcinoma) groups or among the normal, stage IB-IIC1, and stage IIIC2-IVB groups for (A) miR-142-2p, (B) four lncRNAs, (C) five mRNAs, (D) four snoRNAs, (E) four piRNAs, (F) four snRNAs, (G) three tRNAs, and (H) two yRNAs. RPM; reads per million mapped reads, RPKM; reads per kilo base per million mapped reads.
