# Supplementary material for: Screening Plasma Exosomal RNAs as Diagnostic Markers for Cervical Cancer: An Analysis of Patients Who Underwent Primary Chemoradiotherapy
Source: Biomolecules. 2021 Nov 14;11(11):1691. doi: 10.3390/biom11111691 (PMC8615616; doi:10.3390/biom11111691)
Supplement: Supplementary file 1 [file biomolecules-11-01691-s001.zip › Supplementary_method_R1.pdf]

Supplementary Methods

Plasma preparation & storage

In principle, blood was collected in an EDTA tube (purple stopper), stored at 4℃, and centrifuged within 2 h at 13,000 rpm and 4℃ for 10 min. The uppermost yellow layer of the centrifuged blood was divided at 300 μl into sterilized cryotubes and then the tube cap was labeled according to the assigned blood resource number. For long-term plasma storage, the conditions are pp tube, 0.5–2 ml, and (-85)~(-60)℃.

The following paragraphs describe the process of plasma exosomal RNA sequencing

Small RNA library construction & sequencing

Exosome was isolated from human plasma by mixing the plasma with Exo2D RNA solution (Exosomeplus). The detailed isolation process was performed according to the manufacturer's instructions attached separately. RNA from plasma-derived exosomes was extracted using the miRNeasy Serum/Plasma Kit (Qiagen, Valencia, CA) according to the manufacturer's instructions attached separately. The extracted RNA concentration was calculated by Quant-IT RiboGreen(Invitrogen). RNA size was confirmed using Agilent RNA 6000 Pico Kit and Small RNA Kit on Agilent 2100 Bioanalyzer (Agilent Technologies, Böblingen, Germany). The 10ng of RNA isolated from each sample was used to construct sequencing libraries with the SMARTer smRNA-Seq Kit for Illumina, following the manufacturer's protocol. Briefly, Input RNA is first polyadenylated in order to provide a priming sequence for an oligo(dT) primer. cDNA synthesis is primed by the 3' smRNA dT Primer, which incorporates an adapter sequence at the 5'end of each RNA template, it adds non templated nucleotides which are bound by the SMRT smRNA Oligo-enhanced with locked nucleic acid (LNA) technology for greater sensitivity. In the template-switching step, PrimeScript RT uses the SMART smRNA Oligo as a template for the addition of a second adapter sequence to the 3'end of each first-strand cDNA molecule. In the next step, full-length Illumina adapters (including index sequences for sample multiplexing) are added during PCR amplification. The Forward PCR Primer binds to the sequence added by the SMART smRNA Oligo, while the Reverse PCR Primer binds to the sequence added by the 3' smRNA dT Primer. The amplified libraries were purified from 6% Novex TBE-PAGE gels (Thermo Fisher, MA) to excise over than 138 bp (over than 18 bp of cDNA plus 120 bp of adaptors) fraction.

Resulting library cDNA molecules include sequences required for clustering on an Illumina flow cell. The libraries were gel purified, and validated by checking the size, purity, and concentration on the Agilent Bioanalyzer. The libraries were quantified using qPCR according to the qPCR Quantification Protocol Guide (KAPA Library Quantificatoin kits for Illumina Sequecing platforms) and qualified using the TapeStation D1000 ScreenTape (Agilent Technologies, Waldbronn, Germany). The libraries were pooled in equimolar amounts, and sequenced on an Illumina HiSeq 2500 (Illumina,San Diego, USA) instrument to generate 51 base reads. Image decomposition and quality values calculation were performed using the modules of the Illumina pipeline.

Adapter trimming

The raw sequencing reads of small RNAs from the different experimental samples were pre-processed and analyzed with miRDeep2. Adapter trimming processed is done using cutadapt program to eliminate the adapter sequences that exist in the read which were attached to the miRNA during the smRNA library construction process. The first 3nt of all reads were trimmed to remove extra bases inserted during the SMART template-switching activity process. The adapter sequence and everything 3' of the adapter were also removed. If a read matches more than at least first 5 bp of 3' adapter sequence, it was regarded the sequence truly is adapter sequence, and then trimmed from the read. Trimmed reads should be at the minimum of 18 bp in order to be considered reliable for analysis. Then, the remaining reads are classified into non-adapter reads, if they are not sequenced adapter sequences. In this analysis, trimmed and non-adapter reads were combined and regarded as processed reads for downstream analysis.

Clustering

To minimize the sequence uniqueness and computational intensity, adapter sequence processed reads are gathered and form a cluster. This cluster contains reads that are 100% match to the sequence identity and read length and are given its temporary cluster ID and the number of reads it holds.

Ribosomal RNA filtering

Most of the RNA composition is known as rRNA. In order to eliminate the effect of large amounts of rRNA, the read was aligned to the 45S pre-rRNA and mitochondrial rRNA of Homo sapiens and matched.

mRNA expression profiling

The reference gene annotation for *Homo sapiens* (GRCh38; release 109.20190607) was retrieved from NCBI. Since the produced read contains not only small RNA but also mRNA, mRNA expression profiling was performed

using RSEM (v1.3.1) with options (--estimate-rspd --seed-length 15 --strandedness forward).

Identification of known miRNA reads

Sequence alignment and detection of known and novel microRNAs were performed using miRDeep2 software algorithm. Prior to performing sequence alignment, the *Homo sapiens* reference genome was indexed using Bowtie (1.1.2), a bowtie for aligning sequencing reads to reference sequences. Those reads were then aligned *Homo sapiens* matured and precursor miRNAs obtained from miRBase v21. The miRDeep2 algorithm is based on the miRNA biogenesis model; it aligns reads to potential hairpin structures in a manner consistent with Dicer processing, and assigns scores that represent the probability that hairpins are true miRNA precursors. In addition to detecting known and novel miRNAs, miRDeep2 estimates their abundance.

Proportion of miRNA and other RNA categories

Uniquely clustered reads are then sequentially aligned to reference genome, miRBase v21 and non-coding RNA database RNAcentral release 10.0 to identify known miRNAs and other type of RNA.
